# Instability Mechanism in Thermoelectric Mg_2_(Si,Sn) and the Role of Mg Diffusion at Room Temperature

**DOI:** 10.1002/smsc.202300298

**Published:** 2024-02-14

**Authors:** Amandine Duparchy, Radhika Deshpande, Aryan Sankhla, Sanyukta Ghosh, Julia Camut, Sungjin Park, SuDong Park, Byungki Ryu, Eckhard Mueller, Johannes de Boor

**Affiliations:** ^1^ Institute of Materials Research German Aerospace Center (DLR) D–51147 Cologne Germany; ^2^ Energy Conversion Research Center Electrical Materials Research Division Korea Electrotechnology Research Institute (KERI) Changwon 51543 South Korea; ^3^ Institute of Inorganic and Analytical Chemistry Justus Liebig University Giessen Heinrich‐Buff‐Ring 17 D–35392 Giessen Germany; ^4^ Faculty of Engineering Institute of Technology for Nanostructures (NST) and CENIDE University of Duisburg‐Essen 47057 Duisburg Germany

**Keywords:** first‐principles calculations, magnesium diffusion, material stability, Mg_2_(Si,Sn), transport property analysis

## Abstract

Mg_2_(Si,Sn) shows great promise as thermoelectric material as it is made from non‐toxic, abundant, and cost‐effective elements offering high performance. This has been emphasized by several thermoelectric generator prototypes, demonstrating technological maturity. However, material stability is paramount for large‐scale applications whereas we reveal here that the thermal stability of n‐type Mg_2_(Si,Sn) may be limited even at room temperature (RT). Integral thermoelectric properties measurements, locally resolved Seebeck coefficient analysis, scanning electron microscopy/energy‐dispersive X‐ray spectroscopy, and atomic force microscopy are employed to assess changes of n‐type samples stored in ambient atmosphere for years, revealing the evolution of the carrier concentration and transport properties in the material as well as surface degradation. This is caused by the diffusion of loosely bound Mg from the bulk towards the surface and subsequent oxidation, leading to a change of Mg‐based intrinsic defect concentrations, thereby degrading the thermoelectric performance. This microscopic mechanism is backed up by first‐principles calculations, revealing that Mg diffusivity in Mg_2_(Si,Sn) is high at RT and that diffusion occurs mainly via Mg vacancies. The observed much faster degradation of Sn‐rich Mg_2_(Si,Sn) can be correlated with the higher density of Mg vacancies in Mg_2_Sn compared to Mg_2_Si, as predicted from defect formation energies.

## Graphical abstract

This work investigates a multistep material degradation mechanism driven by Mg diffusion within n‐type Mg_2_(Si,Sn) thermoelectrics while stored under ambient atmosphere at room temperature. It involves Mg bulk diffusion as a limiting process step for Mg migration toward the sample surface and subsequent reaction with moisture leading to a pronounced deterioration of the thermoelectric performance.

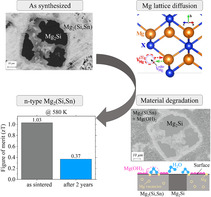

## 1 Introduction

The current energy crisis along with high demand in energy and the need to reduce greenhouse gas emission and replace fossil fuel consumption raises a critical need for new clean and renewable energies.^[^ ^1^, ^2^ ^]^ One of the alternatives would be the use of thermoelectric (TE) generators, which are promising power suppliers for a wide range of applications such as in the automobile industry,^[^ ^3^, ^4^ ^]^ for wearable medical devices,^[^ ^5^, ^6^, ^7^ ^]^ electronic devices,^[^ ^8^ ^]^ or the aerospace industry where radioisotope TE generators have been used for various space missions (Mars Curiosity and Perseverance rovers, Voyager 1 and 2, Cassini to Saturn, to name a few among numerous).^[^ ^8^, ^9^, ^10^ ^]^ Such generators directly convert heat into electricity with the advantages of extremely high reliability due to the absence of moving parts. The efficiency of TE devices itself depends on the performance of materials, governed by the TE figure of merit $\text{zT}=\frac{S^{2}\sigma }{\kappa }T$, where *S* is the Seebeck coefficient, *σ* is the electrical conductivity, *T* is the absolute temperature, and κ is the thermal conductivity. An optimized TE material should have a low thermal conductivity and a high TE power factor PF = *S* ^2^ *σ*.^[^ ^11^ ^]^ The Seebeck coefficient and the electrical conductivity are largely governed by the charge carrier concentration (*n*) and the charge carrier mobility (*μ*), which needs to be optimized.

In the recent years, a significant progress in the TE field has been achieved with the development of highly efficient TE materials (zT > 1) such as PbTe,^[^ ^12^ ^]^ half‐Heusler compounds,^[^ ^13^ ^]^ CoSb_3_‐based skutterudites,^[^ ^14^ ^]^ or Mg‐based TE materials such as MgAgSb,^[^ ^15^, ^16^, ^17^ ^]^ Mg_3_Sb_2_,^[^ ^18^, ^19^ ^]^ or magnesium silicide/magnesium stannide (Mg_2_X with X = Si,Sn) based solid solutions.^[^ ^20^, ^21^ ^]^ Among these materials, Mg_2_(Si,Sn) solid solutions are an attractive and promising material class in the mid‐temperature range (room temperature [RT] to 723 K) due to its light weight, cheap, nontoxic, environmental friendly, and abundant elemental constituents (Mg, Si, and Sn).^[^ ^22^, ^23^ ^]^ Developing low‐cost, nontoxic, and efficient TE materials with good thermal stability is crucial for technological development and TE product implementation in a mass market.

Mg_2_Si–Mg_2_Sn solid solutions exhibit TE performance superior to the binary compounds due to alloy scattering which reduces the lattice thermal conductivity. Zaitsev et al.^[^ ^24^ ^]^ and Zhu et al.^[^ ^25^ ^]^ have also shown that the solid solution transport properties strongly depend on the Si to Sn ratio (*x* parameter in Mg_2_Si_1−*x*_Sn_*x*_). They found that n‐type Mg_2_Si_1–*x*_Sn_*x*_ solid solutions (with *x* = 0.6–0.7) have excellent TE properties (zT_max_ ≈ 1.4) because of a degeneracy of the conduction bands and low lattice thermal conductivity.^[^ ^21^, ^25^, ^26^, ^27^ ^]^ Suitable dopants of these materials are bismuth and antimony. Further improvement might be achieved by exploiting phase separation process to achieve specific beneficial nanostructuring that involves the formation of a locally varying electronic band structure suitable for selective energy filtering of charge carriers.^[^ ^28^, ^29^, ^30^ ^]^ Last but not least, the material system has achieved technological maturity as demonstrated by successful assembly of several Mg_2_(Si,Sn)‐based modules and a recent Mg_2_(Si,Sn)/MgAgSb module, which achieved conversion efficiencies between 3% and 7%.^[^ ^31^, ^32^, ^33^, ^34^ ^]^

However, while the TE properties are attractive and technological progress has been achieved, there are also indications for a limited stability of the material system. There is first the issue of Mg‐loss during the melting and compacting step at high temperature of the synthesis, originating from the high vapor pressure of Mg.^[^ ^35^, ^36^, ^37^ ^]^ Furthermore, Yin et al. have analyzed the effect of heat treatment on Sb‐doped Mg_2_Si_0.3_Sn_0.7_ and concluded that charge and heat transport property deterioration was linked to Mg sublimation.^[^ ^38^ ^]^ Kato et al.^[^ ^39^ ^]^ and Sankhla et al.^[^ ^40^ ^]^ conducted further studies on the role of Mg content for the change in the properties of the material at elevated temperatures. It was shown that electronic transport properties in Mg_2_Si_1–*x*_Sn_*x*_ are very sensitive to Mg loss leading to changes in the majority charge carrier concentration and hence deteriorating the transport properties.^[^ ^36^, ^38^, ^39^, ^40^, ^41^ ^]^ In contrast, recent work by Skomedal et al. focused on the high‐temperature oxidation behavior of Mg_2_(Si,Sn) solid solution. Their study revealed a significant material degradation through oxidation at elevated temperatures, leading to the formation of a non‐protective MgO layer on the surface and the presence of Sn‐rich liquid at the interface between MgO and Mg‐depleted Mg_2_Sn.^[^ ^42^ ^]^ The observed material and transport property changes indicate stability issues of the material at elevated temperatures, which will prevent the successful development and widespread applications of Mg_2_(Si,Sn)‐based TE generators. Addressing these issues therefore stands as one of the major challenges for achieving large‐scale implementation of such generators.

Based on repeated measurements on samples, we find indications that the stability of the material, when stored in ambient environments for months, is limited. Our investigations focus on Mg‐poor p‐type Mg_1.97_Li_0.03_Si_0.3_Sn_0.7_ and Mg‐rich n‐type Mg_2.06_Si_0.3_Sn_0.665_Bi_0.035_ due to their high TE performance and previous implementation in prototype devices.^[^ ^32^ ^]^ The samples were produced via a melting process,^[^ ^43^ ^]^ resulting in the desired primary phase along with a minor amount of Si‐rich Mg_2_Si_1−*x*_Sn_*x*_ evenly distributed within the sample. Even though these Si‐rich phases don't influence the TE properties significantly, they offer an opportunity to investigate the relevance of the Si:Sn ratio on material stability within a single sample. Both n‐ and p‐type samples were then stored at RT for several years in an ambient environment to assess the effects of the storage condition on sample stability overtime. Here, ambient environment means that the atmosphere does not only contain oxygen but also moisture.

First, we compared the microstructure and integral TE properties of n‐ and p‐type samples. Note that the n‐type samples were synthesized using excess Mg while p‐type samples were not. We show that Mg‐rich n‐type samples degrade overtime even at RT (a decrease of charge carrier concentration) while Mg‐poor p‐type samples remain stable. This difference is presumably due to the Mg content that is different between both materials. Locally resolved Seebeck coefficient measurements show increasing inhomogeneity on a microscale for the n‐type samples with increasing storage duration. Lastly, scanning electron microscopy (SEM) combined with energy‐dispersive X‐ray spectroscopy (EDX) and atomic force microscopy (AFM) were used to analyze the microstructural changes at the surface of the samples and reveal the formation of Mg(OH)_2_ on the sample surface with a selective growth on Sn‐rich areas of the sample, while Si‐rich regions remain unchanged. This indicates that the surface degradation is highly selective to the Si:Sn ratio.

This observation is supported by comparative diffusion couple experiments of Mg/Mg_2_(Si,Sn) and Mg/Mg_2_Si, where only for the former a change in carrier concentration (and hence Mg content) is found by microprobe measurements of the Seebeck coefficient. First‐principles calculations of the formation energies of Mg‐related point defects and Mg diffusivities confirm high mobilities of Mg in Mg_2_Si and Mg_2_Sn and identify transport of Mg via Mg vacancies ($V_{\text{Mg}}^{2-}$) as the dominant mechanism. From the analysis of the calculation results, the observed selectivity of Mg diffusion with respect to the Si:Sn ratio can be rationalized by the lower defect formation energies of $V_{\text{Mg}}^{2-}$ in Mg_2_Sn compared to Mg_2_Si, while further reasons for the selectivity are analyzed. This study reveals that loosely bound Mg in n‐type samples can diffuse out of the material already at RT by the following mechanism: 1) lattice diffusion from the inner to the grain boundary to the sample surface and 2) subsequent Mg oxidation at the surface. The findings emphasize the need for controlled storage conditions of Mg_2_(Si,Sn), especially for Sn‐rich materials.

## 2 Results

P‐ and n‐type samples with compositions around Mg_2_Si_0.3_Sn_0.7_ are analyzed and compared in this study. One crucial difference between the n‐type and p‐type materials is the Mg concentration. Even though Mg_2_(Si,Sn) is nominally a line phase, it has a finite solubility range, i.e., all compositions between Mg_2(1 + *δ*1)_(Si,Sn) and Mg_2(1 + *δ*2)_(Si,Sn) (*δ*1 < *δ*2, both can be negative) might be formed. Mg_2(1 + *δ*1)_(Si,Sn) and Mg_2(1 + *δ*2)_(Si,Sn) are the so‐called Mg‐poor and Mg‐rich thermodynamic states, which can be defined by the chemical potential of Mg and are governed by the concentration of intrinsic point defects. There are several Mg‐related intrinsic defects in Mg_2_(Si,Sn), such as Mg interstitials and Mg vacancies with relatively small defect formation energies, as reported in Ryu et al.^[^ ^44^ ^]^. Under the Mg‐rich condition, the Mg interstitial defect is dominant contributing conduction electrons as Mg on the interstitial position $I_{\text{Mg}}^{2+}$ functions as an electron donor. When going from Mg‐rich to Mg‐poor conditions, the defect density of the Mg vacancy increases and its density can be even larger than that of Mg interstitials, depending on *x* in Mg_2_Si_1–*x*_Sn_*x*_. Mg vacancies $V_{\text{Mg}}^{2-}$ are acceptor defects, compensating electrons.^[^ ^44^, ^45^ ^]^ The solubility range of Mg, i.e., *δ*2–*δ*1 was estimated to be ≈10^−3^ by Sankhla et al.^[^ ^40^ ^]^ and Kato et al.^[^ ^39^ ^]^ for Mg_2_Si_0.4_Sn_0.6_ and Mg_2_Si_0.3_Sn_0.7_ (note that *δ*1 and *δ*2 are slightly differently defined there). Mg that is removed from the material when going from the Mg‐rich to the Mg‐poor state is denominated as loosely bound Mg, as it can be removed without destroying the compound. Here (and throughout literature^[^ ^43^, ^46^, ^47^, ^48^ ^]^) largely overstoichiometric amounts of Mg, i.e., Mg_2(1 + *δ*3)_(Si,Sn) with *δ*3 ≈ 0.03 >> *δ*1 are employed to achieve a Mg‐rich composition for n‐type Mg_2_(Si,Sn) despite the losses during the synthesis process. It is plausible that a Mg‐rich state is indeed achieved for the analyzed sample as the properties are quite reproducible and the sample properties don't react sensitively on small, unintended changes in synthesis conditions,^[^ ^46^ ^]^ which would be expected if the sample was compositionally located between Mg rich and Mg poor. If Mg beyond the solubility limit (“excess Mg”) is contained in the sample it might be located, e.g., at the grain boundaries in thermodynamic equilibrium with Mg_2_X and will not be relevant to the electrical properties of the sample when in small amounts. The p‐type is instead synthesized nominally with a 2:1 for (Mg,Li):(Si,Sn), but some Mg loss will occur during the synthesis and Li is known to not only substitute Mg, but also to go on the interstitial position,^[^ ^49^ ^]^ leading effectively to a Mg‐deficient composition and a Mg‐poor state for the p‐type material. The Mg‐related point defects influence the carrier concentration significantly, in addition to Bi, which was employed as extrinsic dopant here. With respect to the thermodynamic state of the n‐type material, we believe that the material still has Mg excess despite the loss of Mg during synthesis as the introduced excess of Mg is likely beyond the solubility limit.^[^ ^39^, ^40^ ^]^

**Figure 1:**
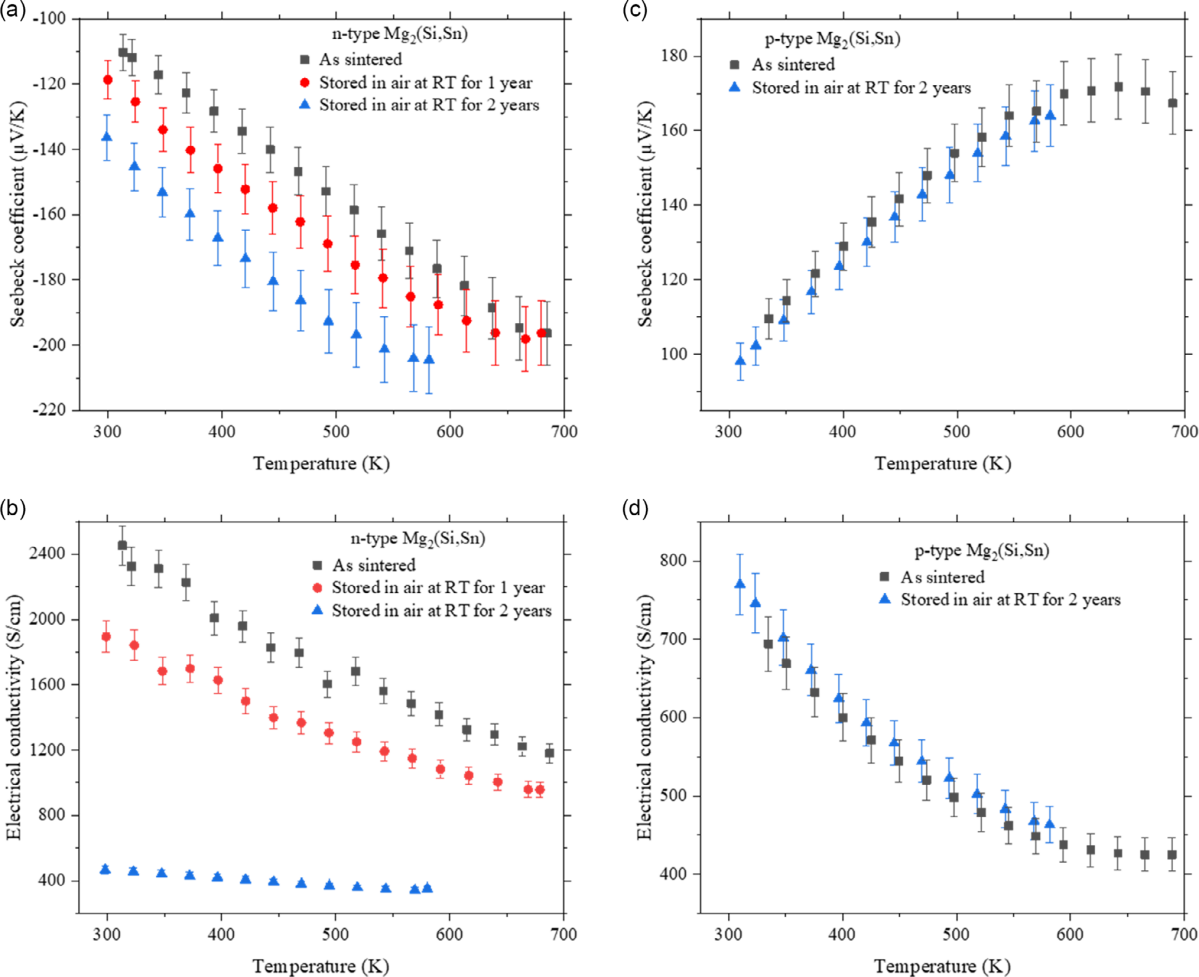
Temperature‐dependent transport properties (Seebeck coefficient and electrical conductivity) of a,b) an n‐type Mg_2_._06_Si_0.3_Sn_0.665_Bi_0.035_ and c,d) a p‐type Mg_1.97_Li_0.03_Si_0.3_Sn_0.7_ sample, directly after synthesis and after being stored in air at RT for several years. Cooling data of the temperature cycle for measurement is plotted and *T*_max_ was restricted for the later measurements to not change the sample due to the measurement conditions themselves. For both n‐ and p‐type, all measurements were conducted on the same sample, without any polishing after aging.

**Figure** **1**a–d illustrates the Seebeck coefficient and the electrical conductivity, of both n‐ and p‐type Mg_2_(Si,Sn) solid solutions immediately after synthesis and after subsequent storage in ambient conditions at RT for a duration of 1 and 2 years for the n‐type and 2 years for the p‐type.

It is evident that before aging, both samples show the typical behavior of a heavily doped degenerate semiconductor with TE properties comparable to those reported in previous studies (PF_n‐type_ = 44 μW cm^−1^ K^−2^ at 590 K here vs PF = 45 μW cm^−1^ K^−2^ at 573 K from ref. [43] for the n type and PF_p‐type_ = 12 μW cm^−1^ K^−2^ at 583 K vs PF = 12 μW cm^−1^ K^−2^ at 573 K from ref. [50]). It demonstrates the high performance of these materials. There are discernible differences in the behavior of the n‐ and p‐type materials upon reevaluation of their transport properties after long storage at RT. In the case of p‐type TE properties, it is evident that they remain stable over a period of 2 years, exhibiting minimal change within the measurement device uncertainty margin of approximately 5%.^[^ ^51^, ^52^ ^]^ Conversely, the n‐type solid solution shows a distinct instability and a substantial degradation of its TE properties with the passage of time. This degradation is manifested by a steady increase in the (absolute) Seebeck coefficient (relative deviation of 20% at RT) alongside a reduction in electrical conductivity at least by a factor of 5 within 2 years. The maximum power factor decreases from 44 to 15 μW cm^−1^ K^−2^ at 573 K, leading to a large decrease in the overall figure or merit, as shown in Figure S1, Supporting Information. Thus, we will focus the rest of the study on the n‐type material to understand the degradation mechanisms.

Comparing the second and the third measurement (Figure 1a,b), it is evident that the transport properties deteriorated significantly, and this deterioration occurred at a notably higher rate as compared to the initial measurement after 1 year. An explanation on the increase of degradation rate could be related to the fact that the sample surface was initially covered by a graphite layer (required for the measurement of the thermal conductivity) which was removed for the measurement after 1 year. Then, the sample remained uncovered during the latter storage period. The observed increase in the Seebeck coefficient and a drastic decrease in the electrical conductivity of the n‐type material overtime can be attributed to variations in the charge carrier concentration, reducing from 3.3 × 10^20^ cm^−3^ for the freshly synthesized sample to 2.2 × 10^20^ cm^−3^ after 2 years of storage in ambient conditions at RT; the values are obtained from the measured Seebeck coefficient assuming a constant effective mass of 2.7*m*_0_ within the parabolic and rigid band approximation, with *m*_0_ being the electron rest mass. This assumption of a constant effective mass is reasonable as was shown by Naithani et al.^[^ ^53^ ^]^ through a single parabolic band (SPB)‐model‐based analysis for a variety of Mg_2_Si_0.3_Sn_0.7_ specimens that the density‐of‐states effective mass does not change with a change of carrier concentration.

**Figure 2:**
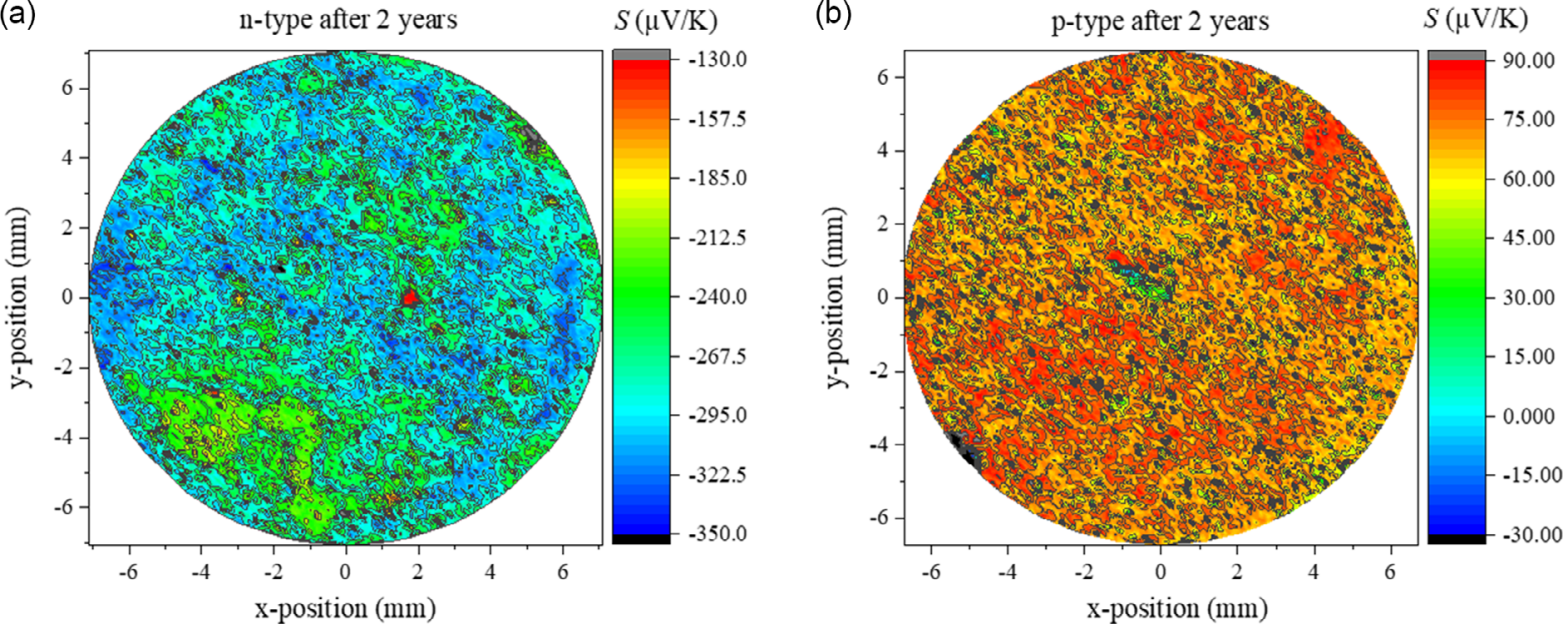
Surface scan of the Seebeck coefficient of stored a) n‐type and b) p‐type Mg_2_(Si,Sn) at RT after 2 years.

A potential and Seebeck microprobe (PSM)^[^ ^54^ ^]^ was utilized to map the Seebeck coefficient of both n‐type and p‐type samples at the surface. Both samples were measured after storage for several years to investigate differences in their homogeneity. The functional homogeneity and dominant carrier type of both n‐ and p‐type samples after storage in air are shown in **Figure** **2**. This data is compared with the surface Seebeck scans of both p‐ and n‐type as‐prepared samples reported in a previous study.^[^ ^43^ ^]^ Note that the author followed the same melting synthesis route and used almost the same composition for the n type (Mg_2.06_Si_0.3_Sn_0.675_Bi_0.025_, i.e., using 3 at% excess of Mg) and the exactly same sample composition for the p type (Mg_1.97_Li_0.03_Si_0.3_Sn_0.7_).^[^ ^43^ ^]^ One can compare our bulk data with the literature as the synthesis process is reproducible and well controlled. The measured Seebeck coefficient on the surface before degradation exhibits high functional homogeneity for both n‐ and p‐type reporting an average Seebeck value of –122 ± 7 and 80 ± 4 μV K^−1^, respectively.^[^ ^43^ ^]^

The Seebeck coefficient measured at the surface of the p‐type material after degradation varies roughly from 60 to 90 μV K^−1^ compared to values varying between 60 and 100 μV K^−1^ for the fresh sample. The p‐type sample thus does not show drastic changes in agreement with the integral transport property measurements (Figure 1c,d). The observed differences between the average PSM result and the bulk data at RT (Figure 1) can be explained by the larger cold finger effect^[^ ^55^ ^]^ (detailed in Experimental Section) on the PSM measurement, which leads to a reduction of the measured absolute values by ≈10–20%. In contrast, it is apparent that the Seebeck coefficient of the n‐type material has different values directly after synthesis and after long‐term storage. The Seebeck surface profile in Figure 2a demonstrates substantial alteration in the Seebeck coefficient (values between −200 and −350 μV K^−1^) as a result of change in the carrier concentration. The PSM is a surface‐sensitive measurement, larger values than obtained by bulk measurements are therefore not surprising and indicate a stronger change at the surface compared to the depth of the sample.

**Figure 3:**
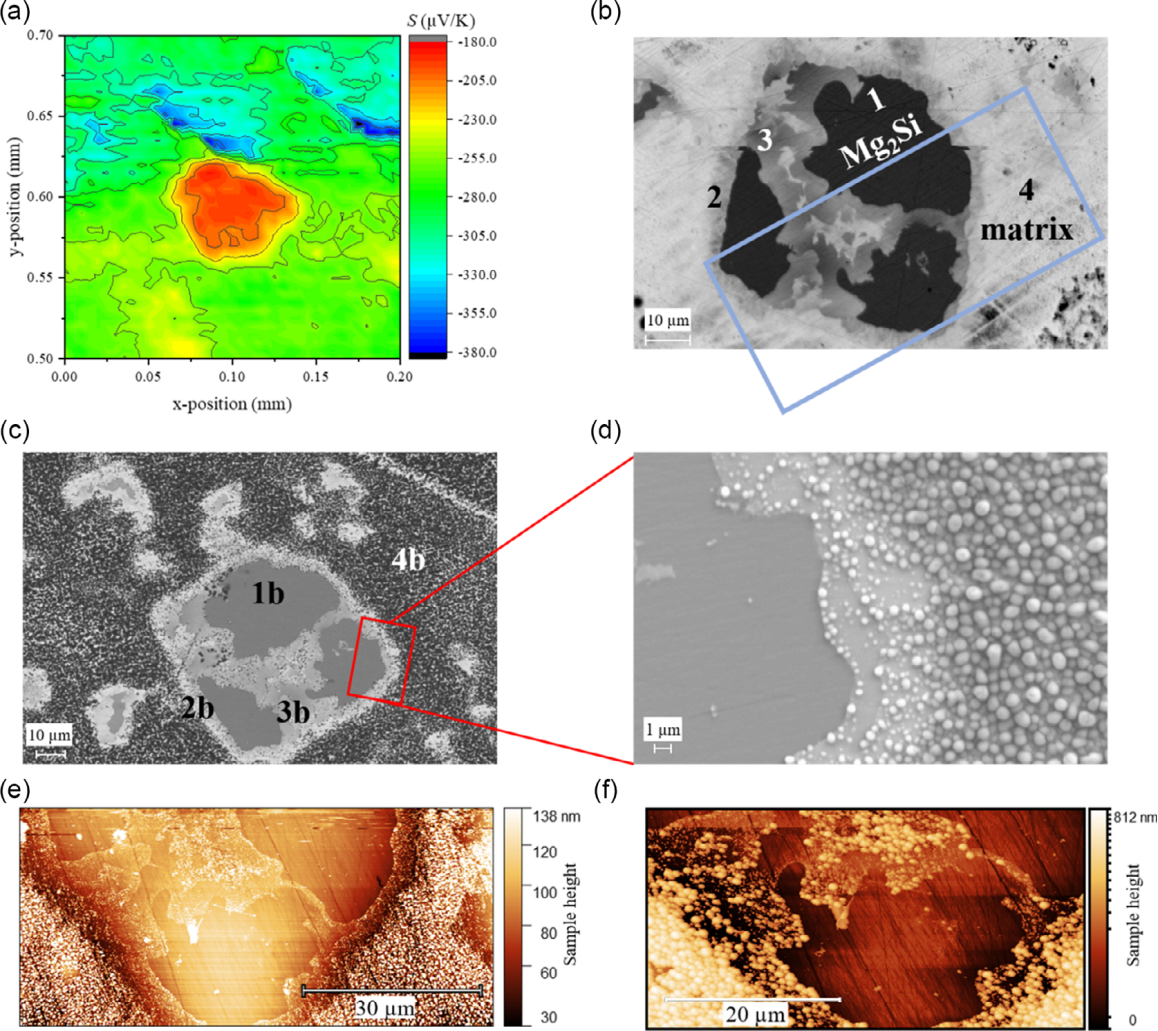
a) Surface Seebeck coefficient map of the n‐type Mg_2.06_Si_0.3_Sn_0.665_Bi_0.035_ sample measured by the transient potential Seebeck microprobe (TPSM) after aging for 2 years of storage at RT, b) its corresponding BSE image of the polished surface. The red area from (a) corresponds to the central feature in (b), dominated by the black phase. c) BSE low magnification image of the unpolished degraded surface of the n‐type sample after 2 years in ambient conditions, showing the same region as in (b); d) secondary electron (SE)‐high magnification image of the unpolished sample. e,f) AFM images of both polished and unpolished surfaces of the n‐type sample. The area in the AFM images corresponds to the region surrounded by the blue rectangle in (b).

A study focused on the local Seebeck coefficient using a transient Seebeck microprobe^[^ ^56^ ^]^ with higher spatial resolution (3–5 μm) was conducted on a small area of the n‐type sample which was slightly polished after being stored in ambient conditions for years, as shown in **Figure** **3**a. An area with a lower value of the Seebeck coefficient can be found (a red spot in Figure 3a). This same area was characterized by SEM–EDX and AFM and is shown in Figure 3c,e, respectively. It corresponds mainly to the black phase visible on the backscattered electrons (BSE) image (Figure 3b), identified as Mg_2_Si, while the rest of the Seebeck map is corresponding to the Mg_2_(Si,Sn) matrix (light gray phase, marked as 4 in Figure 3b). While the samples appear to be single‐phase X‐ray diffraction (XRD)‐wise, such Si‐rich islands are commonly observed in Mg_2_(Si,Sn). They are remnants from the synthesis process and constitute less than 5% of the total volume. Such microstructure is often observed^[^ ^21^, ^40^, ^48^, ^57^, ^58^, ^59^, ^60^, ^61^, ^62^ ^]^ and is probably due to different diffusivities of Si and Sn in Mg_2_(Si,Sn) and the miscibility gap in this material system.^[^ ^29^, ^63^ ^]^ Figure 3d is a zoomed‐in image of the unpolished, degraded surface shown in Figure 3c. Finally, Figure 3b,f represents the BSE image and AFM scan, respectively, of the same spot of the sample after polishing

The large Si‐rich island has a Seebeck coefficient of ≈–200 μV K^−1^, while the Sn‐rich matrix exhibits values around −300 μV K^−1^. Taking the integral Seebeck coefficient measurements and local Seebeck coefficient measurement on fresh samples into account, we can deduce that the Seebeck coefficient of the matrix phase has changed and, assuming a single‐band‐dominated transport, the SPB estimated carrier concentration has reduced significantly from 10^20^ to less than 10^19^ cm^−3^. In contrast, the value of about –200 μV K^−1^ for the Si‐rich phase corresponds to a low carrier concentration as well, but the higher affinity of the Bi‐ to Sn‐rich phase compared to the Si‐rich phase and a recent investigation by Ghosh et al.^[^ ^28^ ^]^ makes it plausible that the carrier concentration of the Si‐rich phase was low also immediately after synthesis, indicating that the matrix is more affected by changes with aging than the Si‐rich island. While differences in the Si:Sn ratio can easily be detected from EDX, changes in Mg and dopant concentrations are below the limit of the resolution of EDX. These changes become distinguishable through the surface Seebeck scans where a difference in the values highlights variations in charge carrier concentration in the matrix after degradation. We offer this region as “polished” microstructure in the SEM images. Although the functional properties have changed during long storage at RT, we observe that the visible microstructure remains the same.

The microstructures of the polished and the degraded, non‐polished surfaces of the same aged sample were investigated using SEM and AFM techniques. Here, the “polished surface” does not correspond to the surface of a fresh sample but of the polished surface of the aged sample. Figure 3b depicts the microstructure of the polished surface after sample degradation while Figure 3d depicts the degraded surface without polishing of the n‐type Mg_2_(Si,Sn) sample that was stored for 2 years in ambient conditions. The micrographs reveal distinctive features both for the polished surface and the degraded surface. An evident‐selective surface degradation phenomenon is observed on the non‐polished surface characterized by the presence of a discernible fourth phase (denoted with “4b” in Figure 3c). The secondary electron image in Figure 3d shows that this new phase has a spherical structure with a size distribution between 0.5 and 1 μm. SEM/EDX analysis of these spherical structures revealed that those are rich in Mg and O. Considering the atomic percent of O, one could conclude that the newly formed phase covering the Sn‐rich main phase of the sample is MgO or Mg(OH)_2_, this being discussed later. One should note that the absolute values of the oxygen concentration are quite inaccurate and only large changes are significant. Hence, the numbers shown in **Table** **1** are only for qualitative comparison. Upon comparing the compositions of phases 1, 2, and 3 for polished and unpolished surfaces (as presented in Table 1), it is apparent that the black phase (phase 1), identified as Mg_2_Si in both cases, remains unaffected and basically unaltered by oxidation. Phases 2 and 3 remain similar with some amount of additional O. Last but not least, Figure 3e,f represents AFM images of both non‐degraded and degraded surfaces of the n‐type sample after aging. The Mg_2_Si phase appears flat in both images, while the thickness of newly formed O‐rich phase 4b in Figure 3d is of the order of several hundred nanometers, covering the Sn‐rich Mg_2_(Si,Sn) matrix. It is important to note that after slight polishing, we don't find any indications for elemental Si or (Mg,Sn) as indicators for a complete decomposition of Mg_2_(Si,Sn), as was shown in experimental investigations of the phase constitution of Mg_2_(Si,Sn) annealed at high temperature in the context of the equilibrium phase diagram.^[^ ^29^, ^64^, ^65^ ^]^ It is plausible that phase 4 is formed by the reaction of air or moisture with loosely bound Mg. Loosely bound Mg might be present in both Sn‐rich and Si‐rich areas, but moisture/air seems to react only with the Mg of Sn‐rich phases. These observations suggest that Si‐rich secondary phases exhibit greater stability against oxidation and/or lower Mg diffusion at RT.

**Table 1:** Phase compositions in n‐type Mg_2.06_Si_0.3_Sn_0.665_Bi_0.035_ calculated from EDX analysis of the different phases found before and after polishing

Phases	Degraded, unpolished					After polishing				
	Mg	Si	Sn	Bi	O	Mg	Si	Sn	Bi	O
Phase 1 [at%]	64.3	30.5	1.8	0.1	3.3	65.9	29.9	2.3	0.1	1.8
Phase 2 [at%]	57.8	15.5	6.9	0.5	19.3	64.5	18.2	13.8	1.0	2.5
Phase 3 [at%]	61.4	23.6	4.2	0.3	10.5	65.7	24.2	8.1	0.4	1.6
Phase 4 [at%]	28.7	2.7	4.7	0.25	63.7	63	7.9	23.6	1.1	4.4

**Figure 4:**
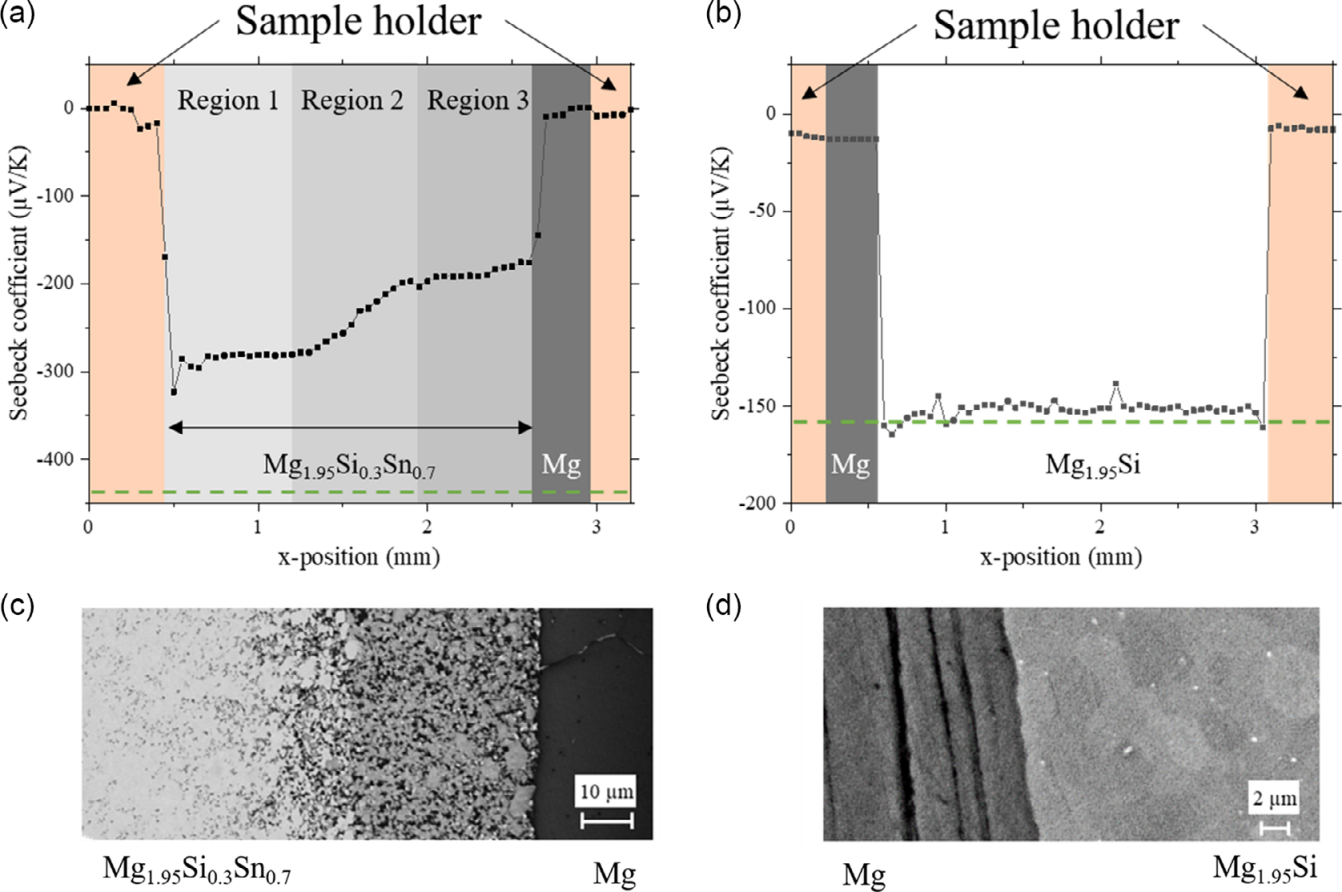
Representative Seebeck coefficient profiles for a) Mg_1.95_Si_0.3_Sn_0.7_/Mg diffusion couple and b) Mg_1.95_Si/Mg diffusion couple. Both diffusion couples were joined at 823 K for 20 min. The dark gray rectangle indicates Mg foil, the orange rectangles are the sample holder made of Cu and the lighter gray rectangles in (a) delimit diffusion regions. The horizontal dashed green line indicates the value of the sample's Seebeck coefficient before joining with Mg. c,d) The BSE images of the respective interfaces. It is worth pointing to the difference in scale between (a–c) and (b–d) that is in the order of millimeter for the Seebeck scans and of micrometer for the SEM images, respectively. This means that the 20 μm thick layer forming adjacent to Mg in (c) is not visible in the PSM line scan.

Diffusion couple experiments were conducted to investigate Mg diffusion in Mg_2_(Si,Sn) further. Two diffusion couples, one of Mg‐deficient Mg_1.95_Si_0.3_Sn_0.7_ joined with Mg and a second of Mg_1.95_Si also joined to Mg were prepared at 823 K. It is reasonable to correlate changes at RT over long durations with short high‐temperature experiments as higher temperature accelerates diffusion processes. RT Seebeck line scans of Mg/Mg_1.95_Si_0.3_Sn_0.7_ and Mg/Mg_1.95_Si are presented in **Figure** **4**a,b, respectively. The PSM line scans are an excellent tool to probe microscale changes in intrinsic or extrinsic defect concentrations, caused, e.g., by the diffusion of dopants or in our case, Mg^[^ ^44^, ^66^, ^67^ ^]^ due to its sensitivity to carrier concentration.

The solid solution sample joined with Mg as shown in Figure 4a displays an interesting Seebeck profile where the absolute values of Seebeck coefficient decrease near the interface and reach a saturation at −300 μV K^−1^. The value near the interface (Region 3) is reduced to −175 μV K^−1^. The Seebeck coefficient value of the bulk Mg_1.95_Si_0.3_Sn_0.7_ was initially −450 μV K^−1^ as indicated by the green line and changed to −300 μV K^−1^ (Region 1) after joining. Large absolute values for *S* with respect to the changes from −450 to −300 μV K^−1^ correspond to low carrier densities, i.e., nearly complete compensation between vacancies and interstitials. In this regime, the Seebeck coefficient reacts sensitively to small changes, which might be induced by a relaxation of intrinsic defects due to the thermal treatment related to the joining step, which is at lower temperature than the initial compaction process of the sample. With respect to the previously discussed role of Mg‐related defects on the carrier concentration,^[^ ^39^, ^40^ ^]^ one can deduce that the different regions of the diffusion couple correspond to regions with different defect concentrations, i.e., different Mg content. Close to the Mg foil, Mg atoms dissolved from the foil into the semiconductor will compensate the vacancies in the Mg‐depleted Mg_1.95_Si_0.3_Sn_0.7_ and thus shift the balance between vacancies and interstitials, increasing the n‐type carrier density. As a result, three diffusion regions in the TE material marked with gray rectangles in Figure 4a can be depicted as Region 1 (Mg‐poor region): intrinsic Mg_2_(Si,Sn) with a relatively large density of $V_{\text{Mg}}^{2-}$; Region 2 (linear region): vacancies increasingly compensated by Mg interstitials; and Region 3 (Mg‐rich region): $V_{\text{Mg}}^{2-}$ compensated by $I_{\text{Mg}}^{2+}$ with $I_{\text{Mg}}^{2+}$ being dominant. As Mg interstitials are dominant, this region shows the strongest heavily doped n‐type behavior among the three distinct regions. Note that this indicates that Mg has diffused over milimeter(s) and into the grains of the material to become electrically effective as a dopant. In the Mg/Mg_2_Si diffusion couple, the dominant defect will be $I_{\text{Mg}}^{2+}$ even for Mg‐poor chemical potential, as reported by Ryu et al.^[^ ^46^ ^]^. Thereby, the carrier density will be determined by Mg interstitials. Going from Mg‐poor to Mg‐rich should in principle increase the carrier concentration (at a lower level than for Mg_2_Sn) and hence decrease the Seebeck coefficient. That this is not observed is a clear indication that diffusion of Mg into the grains is negligible in Mg_2_Si under the tested conditions. Temperature‐dependent electrical transport properties of both, bulk Mg_1.95_Si and Mg_1.95_Si_0.3_Sn_0.7_, are plotted in Figure S5, Supporting Information, supporting the fact that the Seebeck coefficient close to Mg is unchanged for Mg_1.95_Si and changed for Mg_1.95_(Si,Sn). From the temperature‐dependent Seebeck coefficient *S*(*T*) of the undoped Mg_2_Si sample, we calculated the reduced chemical potential (*η*), showing that Mg_1.95_Si material is slightly higher doped compared to Mg_1.95_Si_0.3_Sn_0.7_ solid solution. This is in line with the first‐principles study from Ryu et al.^[^ ^46^ ^]^ who showed that for Mg‐poor Mg_2_Si synthesized at higher temperature, Mg_2_Si has a slightly larger carrier density *n* than Mg_2_Si_0.3_Sn_0.7_. Mg‐poor Mg_2_Si_0.3_Sn_0.7_ is very close to the compensation point, where the different defect types compensate to an overall low carrier concentration. Hence, this explains the different observed *n* and the observed difference between bulk and PSM after joining.

BSE images presented in Figure 4c,d illustrate the interface between the TE material and Mg foil. TE material, Mg, and interdiffusion zone are visible in the Mg_1.95_Si_0.3_Sn_0.7_/Mg bar (Figure 4c) whereas Figure 4d displays a sharp interface at the junction of Mg_1.95_Si with Mg. It can be inferred that the bonding process of Mg_1.95_Si_0.3_Sn_0.7_ to Mg leads to the formation of several distinct features. The interface between Mg_1.95_Si and Mg region is flat and there is no visible indication for a chemical reaction between the TE material and the Mg foil. This is also confirmed by the EDX line scan depicted in Figure S3, Supporting Information.

**Figure 5:**
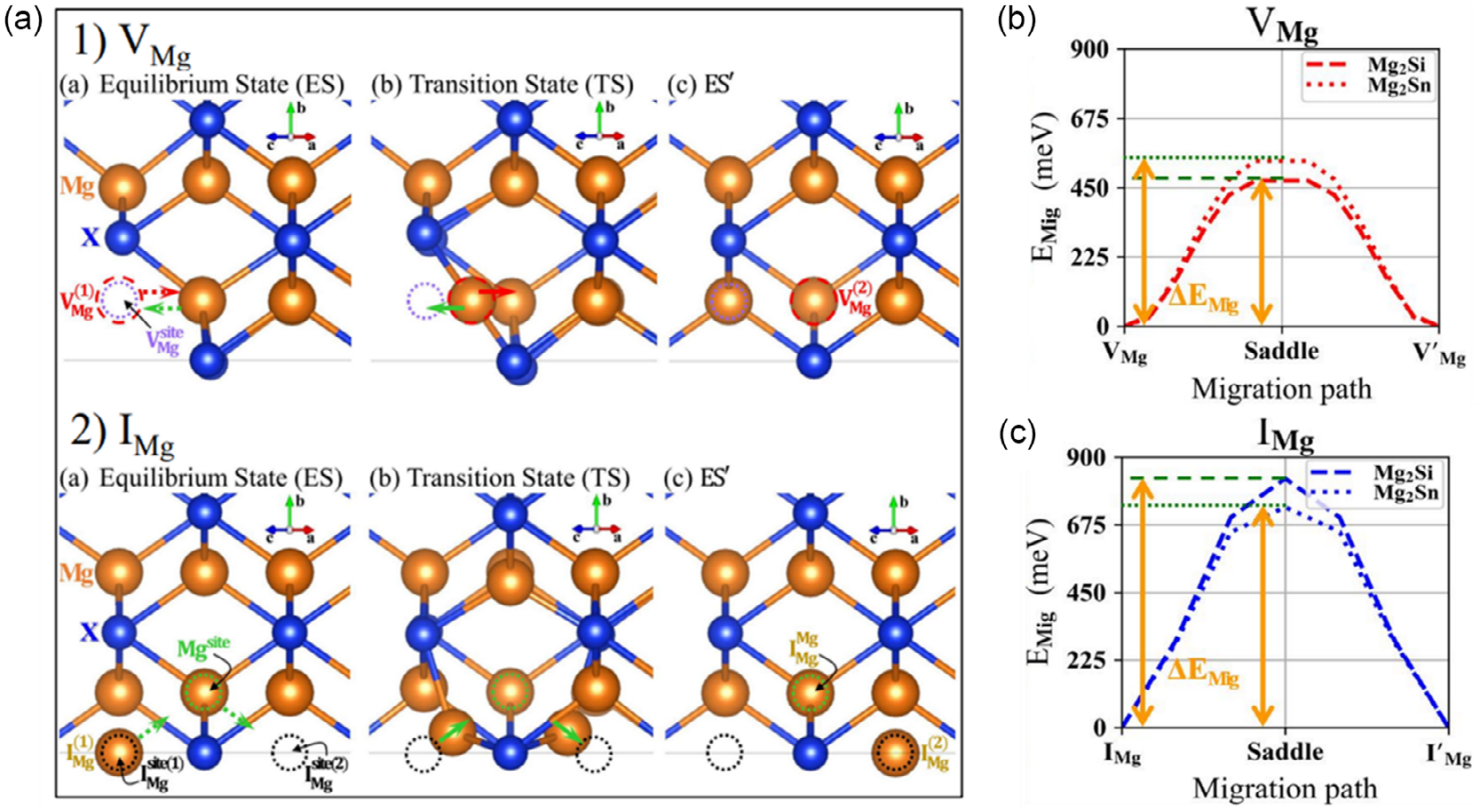
a) Migration path of Mg vacancies ($V_{\text{Mg}}^{\text{2‐}}$) and Mg interstitials ($I_{\text{Mg}}^{\text{2+}}$); b,c) migration energy barrier (*E*_Mig_) for $V_{\text{Mg}}^{\text{2‐}}$ and $I_{\text{Mg}}^{\text{2+}}$ in Mg_2_Si and Mg_2_Sn, respectively.

We performed first‐principles calculations to investigate the diffusion characteristics of intrinsic defects in Mg_2_(Si,Sn) and Mg_2_Si within hybrid‐density‐functional theory (hybrid DFT). The diffusion can occur through different mechanism such as vacancy (**Figure** **5**a‐1), interstitial (Figure 5a‐2) or substitutional diffusion. The defect formation energy (energy required to create a defect in the crystal lattice) and migration barrier (energy which the atom needs to overcome to move from one site to another) are crucial in determining the ease with which diffusion can occur in the material. Note that the defect formation energy is closely related to the defect concentration and hence a lower vacancy formation energy for Mg‐poor material corresponds to high vacancy densities.^[^ ^46^ ^]^ In contrast, the ease of Mg removal is related to the chemical potential of Mg, which is high in Mg‐rich conditions (easy removal) compared to Mg‐poor conditions. We use the climbed‐nudged elastic band (cNEB) method to calculate the migration barriers (*E*_Mig_), for Mg interstitial and Mg vacancy in Mg_2_Si and Mg_2_Sn. Figure 5 shows the diffusion path of Mg interstitials and Mg vacancies in binary Mg_2_X (*X* = Si, Sn). For the diffusion of Mg via interstitials, a Mg atom at the interstitial site replaces a neighboring lattice Mg atom while the “original” Mg atom moves toward a next neighboring Mg interstitial site. With this process, the Mg interstitial defect migrates with migration barriers of 0.828 and 0.734 eV in Mg_2_Si and Mg_2_Sn, respectively. In the case of vacancy migration, a neighboring Mg atom just compensates for the Mg vacancy, generating a new Mg vacancy next to the original Mg vacancy site. For this simple vacancy diffusion path, the migration barriers are 0.472 and 0.537 eV in undoped Mg_2_Si and Mg_2_Sn, respectively, which are much smaller than the barriers for Mg interstitials. We therefore reveal that Mg defects all have small diffusion barriers (less than 1 eV), indicating a possible Mg transport in Mg_2_(Si,Sn).

In an ideal situation, when there are no defect–defect interactions, the interstitials and vacancies can easily diffuse without forming other defect complexes. Substitutional defects can diffuse if there exists a neighboring vacancy, and the diffusion barrier is the sum of vacancy formation energy and defect migration energy. However, for interstitials and vacancies, the diffusion barrier energies are equal to the defect migration energies, as the defects just move with no help of additional defects. Following Fick's law, the defect diffusion can be quantified as the relation between concentration gradient (∇*n*) and defect flow (*J*_Defect_) as *J*_Defect_ = −*D*∇*n* with the diffusion coefficient $D=a_{0}^{2}C_{v}\Gamma$. Here, $a_{0}$ is the jumping distance, $C_{v}$ is unity for vacancy and interstitial diffusion as no formation of an additional defect is required, and $\Gamma =\upsilon^{*}exp\left(-\frac{E_{\text{Mig}}}{k_{B}T}\right)$ is the successful atom jump frequency of interstitials or vacancies where $\upsilon^{*}$ is the effective frequency of a defect with the defect migration energy of *E*_Mig_. From the defect supercell vibration energy distribution, we extract the effective frequency, $\upsilon^{*}$, for defects. The frequencies are calculated as 4.3 and 9.5 THz for Mg interstitial and Mg vacancy in Mg_2_Si, respectively. They are 8.9 and 16 THz in Mg_2_Sn. Then, we calculate the diffusion pre‐factor Γ and the diffusion coefficient *D* at RT of 300 K. Since *D* will be calculated using only *E*_Mig_ and the vibrational frequencies of $V_{\text{Mg}}^{2-}\;$ and $I_{\text{Mg}}^{2+}$, the *D* values won't depend on the electron chemical potential conditions of the material. At RT, for Mg vacancies in Mg_2_Si, *D* is 1.1 × 10^−14^ m^2^ s^−1^ while in Mg_2_Sn, *D* is 1.8 × 10^−15^ m^2^ s^−1^. With this, we can estimate the Mg diffusion length (L) both via Mg interstitials and Mg vacancies in Mg_2_Si and Mg_2_Sn using $L=\sqrt{6Dt}$. Within 100 days, Mg can diffuse 0.7 and 7 μm in Mg_2_Si and Mg_2_Sn via interstitials at RT, respectively, indicating that Mg interstitial is more mobile in Sn‐rich than in Si‐rich Mg_2_(Si,Sn). However, the diffusion length of Mg vacancy is much longer than the one of Mg interstitials with Mg vacancies diffusing 700 and 300 μm in Mg_2_Si and Mg_2_Sn. Irrespective of numerical inaccuracies and simplifications of the model, this indicates that Mg can diffuse easily in Mg_2_Si and Mg_2_Sn, if there is a sufficiently high concentration of vacancies. It is essential to mention here that a long diffusion length does not automatically mean a strong material flow as this is proportional to the concentration of the defects. Long *L* means only that the available defects distribute over a wide area. But with a low defect concentration, little Mg will be transported. As the crystal structures of the Mg_2_(Si,Sn) solid solutions are the same as Mg_2_Si and Mg_2_Sn, the same diffusion mechanisms will take place and high diffusivity of Mg in Mg_2_(Si,Sn) is also plausible. Possibly, lattice distortion due to alloying might lower the migration energy and facilitate diffusion.

## 3 Discussion

The n‐ and p‐type Mg_2_(Si,Sn) samples showing excellent TE properties after sintering have been stored in ambient air at RT for several years. The analysis of transport properties and microstructure revealed that the p‐type (Mg‐deficient) sample remains stable overtime in ambient conditions, whereas the n‐type (with nominal Mg‐excess and plausibly Mg‐rich) sample undergoes significant deterioration: decrease in electrical conductivity, increase in Seebeck coefficient, and increasing mesoscopic inhomogeneity, all presumably caused by diffusion of Mg from the bulk of the sample to the surface, where Mg is oxidized, leading to the observable surface covering. We discuss here two main factors which determine the n‐type material degradation mechanism: the effect of Mg content and Mg diffusion which seems dependent on the Si:Sn ratio.

The experimental results for the n‐type samples of this work can be compared directly with Sankhla et al.'s investigation on the effect of Mg in Mg_2.06_Si_0.385_Sn_0.6_Sb_0.015_ at high temperature.^[^ ^40^ ^]^ First of all, we compare the temperature at which the maximum Seebeck coefficient is reached after sintering and after several years. For the as sintered sample, *S*_max_ is reached at 723 K while *S*_max_ for the sample after 2 years is obtained at 573 K. The same trend was observed after annealing at high temperature by Sankhla et al.^[^ ^40^ ^]^ where the maximum Seebeck coefficient value before annealing was reached at higher temperature than the one after annealing. Furthermore, the change of slope of the electrical conductivity versus temperature (*σ*(*T*) getting flatter) was also observed after annealing Mg_2_(Si,Sn) n‐type material at 710 K in the same study. Regarding the charge carrier concentration and charge carrier mobility, it is worth noting that prior research by Kato et al.^[^ ^39^ ^]^ and Sankhla et al.^[^ ^40^ ^]^ has established the sensitivity of Mg_2_(Si,Sn) solid solution to Mg loss at high temperatures, where the observed changes in Seebeck coefficient and electrical conductivity are explained by Mg loss and the consequent changes in the concentration of charged Mg‐related defects.^[^ ^36^, ^40^ ^]^ As the evolution of *S* and *σ* with temperature after aging in air at RT shows a similar behavior to what was described by Sankhla et al.^[^ ^40^ ^]^ for a high‐temperature‐treated sample, one can conclude that the decrease in charge carrier concentration in the n‐type Mg_2_(Si,Sn) solid solution at RT can be correlated with variations in magnesium content, and this dynamic behavior of the TE properties underscores the material's sensitivity to magnesium loss at RT over a long period of time. Furthermore, it is clear that with time in ambient conditions, the Seebeck coefficient increases, the charge carrier thus decreases and the mobility decreases, as shown in **Table** **2**. For lower carrier concentrations, one might in principle expect a higher mobility, due to less acoustic phonon scattering (due to less high‐energy carriers) and reduced carrier–carrier scattering. However, in our case, the opposite is observed, indicating the formation of additional scattering centers with time. This agrees with the proposed Mg diffusion out of the sample, creating an increasing number of Mg vacancies, which trap a significant fraction of the electrons provided by Bi doping and thus lead to a reduced carrier concentration and dopant efficiency as well as reduced mobility due to point defect scattering of charge carriers. Preliminary analysis of the data of the as sintered and the aged sample showed that the as sintered sample could be well represented by an SPB model considering acoustic phonon scattering and alloy scattering (comparable to Sankhla et al.^[^ ^35^ ^]^), while for the sample after 2 years of storing *σ*(*T*) cannot be reproduced properly, even if grain boundary scattering (as done by Sankhla et al.^[^ ^35^ ^]^) is included as additional scattering mechanism. This indicates that a further scattering mechanism is relevant, potentially point defect scattering due to increased defect concentrations.

**Table 2:** Room temperature transport properties: Seebeck coefficient (*S*), electrical conductivity (*σ*), charge carrier concentration (*n*), and charge carrier mobility (*μ*) of the n‐type Mg_2_._06_Si_0.3_Sn_0.665_Bi_0.035_ sample and p‐type Mg_1.97_Li_0.03_Si_0.3_Sn_0.7_ as sintered and after being stored in ambient atmosphere for 2 years. The charge carrier concentration and mobility have been determined using a Pisarenko plot assuming an SPB‐mediated transport, respectively, with the assumption of a constant effective mass of 2.7*m*_0_ for the n‐type material and 1.5*m*_0_ for the p‐type material.^[^ ^50^ ^]^

Sample type	Storing condition	*S* [μV K^−1^]	*σ* [S cm^−1^]	*n* [10^20^ cm^−3^]	*μ* [cm^2^ V^−1^s^−1^]
n‐type	Samples as sintered	−110	2452	3.3	46.9
	Sample stored in air after 2 years	−136	463	2.2	13.3
p‐type	Samples as sintered	100	740	3.9	11.9
	Sample stored in air after 2 years	100	741	3.9	11.9

SEM and AFM analysis on the n‐type sample after degradation show the presence of a newly formed phase containing Mg and O at the surface of the matrix phase, which remained undefined so far. Indeed, samples synthesized using the melting route are less homogeneous compared to those produced using mechanical alloying^[^ ^37^ ^]^ resulting in Si‐rich inclusions within the Sn‐rich matrix of the sample. Mg–Sn bonding strength is weaker compared to Mg–Si as shown by Kasai et al.^[^ ^68^ ^]^ indicating that Mg_2_Sn is structurally less stable, and if sufficient oxygen or moisture is available in the surroundings of the sample, excess Mg (beyond the solubility limit) and loosely bound Mg might diffuse toward the surface and react with the surrounding atmosphere, leading to the formation of a non‐protective layer at the surface. This is in line with the observed degraded surface (Figure 3d,e), which happens only on Sn‐rich phases (i.e., the matrix) while the Si‐rich phase remains unchanged. Skomedal et al. observed formation of MgO after high‐temperature annealing in air, showing an instability of Mg_2_(Si,Sn) with respect to oxidation.^[^ ^42^ ^]^ However, Li et al.^[^ ^69^ ^]^ studied the RT chemical stability of Mg_3_Sb_2_, which is also a class of TE materials sensitive to Mg‐related charged intrinsic defects, in detail. They showed that the samples stored in ambient conditions degrade by the formation of Mg(OH)_2_, not MgO.^[^ ^69^ ^]^ In our study, the observations from SEM and AFM can be substantiated by comparing the Gibbs free energy of formation (Δ*G*_f_) of MgO and Mg(OH)_2_ at 298 K to determine which phase formation will be energetically favored. Two plausible RT degradation reactions might be written as follows:

Reaction 1:
(1)

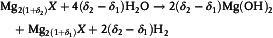

where *δ*_2_–*δ*_1_ is the loosely bound Mg removed from the sample by the formation of $V_{\text{Mg}}^{2-}$ (and reduction of $I_{\text{Mg}}^{2+}$) and diffusion of Mg toward the surface.

Reaction 2:

(2) $${\text{Mg}}_{2\left(1+\delta_{2}\right)}X+(\delta_{2}-\delta_{1})O_{2}\to {\text{Mg}}_{2\left(1+\delta_{1}\right)}X+2(\delta_{2}-\delta_{1})\text{MgO}$$

Note that in both reactions 1 and 2, only loosely bound Mg is consumed, i.e., until the lower solubility limit of Mg in Mg_2_X is reached and that Mg_2_(Si,Sn) does not decompose into elemental components. According to standard thermodynamic data at 298 K, $\left|\Delta G_{f}^{\text{Mg}\left(\text{OH}\right)2}=-834\text{kJ}\;{\text{mol}}^{\text{‐1}}\right|>\left|\Delta G_{f}^{\text{MgO}}=-569\text{kJ}\;{\text{mol}}^{\text{‐1}}\right|$, indicating a stable formation of Mg(OH)_2_ over MgO.^[^ ^70^ ^]^ According to Tan et al. it is well established that MgO only forms at high temperatures,^[^ ^71^ ^]^ and at low temperatures rather Mg(OH)_2_. Furthermore, the hygroscopic nature of MgO is well known and therefore, at RT, Mg(OH)_2_ will be formed (if H_2_O is available) as the more stable form of compound.^[^ ^72^ ^]^ Finally, as MgO is white while Mg(OH)_2_ is black,^[^ ^72^ ^]^ the black appearance of the layer formed on the surface of the sample after years is also a sign that Mg(OH)_2_ develops rather than MgO. All in all, we can thus conclude that the layer that forms at the surface of the matrix is Mg(OH)_2_.

The visible change in the bulk transport properties and the amount of formed Mg(OH)_2_ indicates that Mg must have diffused ≈100 μm at RT following exposure to ambient air. An additional factor impacting the Mg diffusion is the apparent selectivity of Si versus Sn. As suggested previously, diffusion mediated loss of Mg seems to predominantly occur within the Sn‐rich phases. This information is substantiated by the SEM images (refer to Figure 3b–f), surface Seebeck coefficient profiling (see Figure 3a), and the Mg diffusion couples experiments (see Figure 4) where the latter showed that Mg diffuses fast at high temperatures in Sn‐rich Mg_2_(Si,Sn) while no indications for Mg diffusion in Mg_2_Si are found. A difference in the vacancy concentration in Si‐ and Sn‐rich Mg_2_(Si,Sn) could be the reason behind the observed oxidation and alterations in the TE properties exclusively within the matrix, with no or lower impact on the Si‐rich phases.

From the first‐principles calculations, we may explain the changes in the local Seebeck coefficient and hence of carrier concentration of the Si‐rich islands versus the Sn‐rich matrix. We have shown that for $V_{\text{Mg}}^{2-}$ diffusion as the dominant mechanism in Mg_2_Si and Mg_2_Sn, similar diffusivities are obtained. The charged defect formation energy depends on the electron chemical potential, but if this is the same, the vacancy formation is much easier in Mg_2_(Si,Sn). Thus, as the defect density is related to the Boltzmann factor of a given defect formation energy, the defect density of Mg vacancy might be much larger in Sn‐rich than in Si‐rich Mg_2_(Si,Sn). In our case, as Mg transport is proportional to $V_{\text{Mg}}^{2-}$ density, there will be more Mg transport in Sn‐rich Mg_2_(Si,Sn). This indicates that the Mg‐chemical potential will move further toward Mg‐poor conditions as Mg can be lost by the generation of Mg vacancies.

In the case of a Mg‐poor Mg_2_(Si,Sn)/Mg diffusion couple, formation of Mg interstitials seems to be responsible for the change of Seebeck profile, as seen in the linear region (Region 2 in Figure 4a). We predict that Mg‐related defects, vacancies, and interstitials annihilate in this region. Due to the defect interaction, the Mg flow might be smaller than the independent case values. As the Mg vacancies mobility is high, the concentration profile will be a quasi‐steady state showing linear change. First‐principles calculations have shown that Mg vacancies are very mobile, in both Mg_2_Si and Mg_2_Sn, being the main responsible for transport of Mg. Furthermore, in **Table** **3**, we report the formation energies for $V_{\text{Mg}}^{2-}$ in Mg_2_Si and Mg_2_Sn, showing that we have a larger $V_{\text{Mg}}^{2-}$ density in Mg_2_Sn. The Mg transport will be proportional to the Mg mobility (related to the diffusion coefficient) and the defect density that causes the Mg transport (here, $V_{\text{Mg}}^{2-}$). Thus, there will be more Mg transport in Mg_2_Sn than in Mg_2_Si and more where the vacancy concentration is higher.

**Table 3:** Defect formation energies (*E*_Form_) for Mg vacancies ($V_{\text{Mg}}^{\text{2‐}}$) and Mg interstitials ($I_{\text{Mg}}^{\text{2+}}$) in Mg_2_Si and Mg_2_Sn. The formation energies are given for the Fermi level at the conduction band minimum, representative of the highly n‐type‐doped Mg_2.06_Si_0.3_Sn_0.665_Bi_0.035_ sample, but not the diffusion couples

	*E*_Form_ [eV]			
	Mg_2_Si		Mg_2_Sn	
	Mg‐rich	Mg‐poor	Mg‐rich	Mg‐poor
$V_{\text{Mg}}^{2-}$	0.84	0.55	0.76	0.35
$I_{\text{Mg}}^{2+}$	1.08	1.37	0.67	1.08

We hypothesize that loosely bound Mg diffuses toward the surface and oxidizes there, but not Mg formed by the decomposition of Mg_2_X, based on not finding elemental products which would be the result of a complete decomposition of Mg_2_(Si,Sn) at RT. This also naturally explains the observed higher stability of the p‐type material compared to the n‐type material. The main differences are not the diffusivities, which are different but comparable, but the fact that there is no excess Mg in the p‐type material due to employing less Mg in the synthesis. So, while n‐type Mg_2_(Si,Sn) with loosely bound Mg can release Mg by creating $V_{\text{Mg}}^{2-}$, this is not possible for the p‐type material, synthesized without Mg excess.

To sum up, the proposed diffusion mechanism and the calculations explain most of the experimental features for the degradation of Mg_2_(Si,Sn) at RT, including the visible selectivity with respect to doping and employed Si:Sn ratio, at least semiquantitatively. However, we believe that the employed model (isolated, noninteracting defects, single crystal) is oversimplified and there might be further aspects that have not been addressed so far in detail here.

One could also consider the contribution of defect clusters to the Mg transport. So far, using DFT calculations, we have considered isolated defects, in particular Mg vacancies ($V_{\text{Mg}}^{2-}$) and Mg interstitials ($I_{\text{Mg}}^{2+}$). As the Bi dopant at Si/Sn sites is positively ionized while the Mg vacancies are negatively charged, there are attractive electrostatic interaction to form defect clusters like ${\text{Bi}}_{X}^{1}-V_{\text{Mg}}^{2-}\;$ or ${\text{Bi}}_{X}^{1}-V_{\text{Mg}}^{2-}-{\text{Bi}}_{X}^{1}$. The formation of ${\text{Bi}}_{X}^{1}-V_{\text{Mg}}^{2-}$ clusters would be energetically favorable, and there is higher likelihood for charge compensation than considering only isolated defects. Similarly, defect clustering and cluster formation was reported theoretically in Sb‐doped Mg_2_Si_0.6_Sn_0.4_,^[^ ^73^ ^]^ Ag‐doped SnTe,^[^ ^74^ ^]^ and Bi‐doped PbTe.^[^ ^75^ ^]^ But the role of such clusters on Mg diffusion is unclear: if Bi is immobile, they might slow down diffusion due to the trapping and localization of $V_{\text{Mg}}^{2-}$. In contrast, if ${\text{Bi}}_{X}^{1}$–$V_{\text{Mg}}^{2-}$ clusters are also mobile and/or if they reduce the migration barriers for diffusion of Mg via vacancies, they could enhance Mg diffusion. Furthermore, the role of clusters on the TE properties has been highlighted by Kato et al. where it is mentioned that the effect of such defect clusters was prominent for heavily doped specimens, at least beyond 2 at% of Sb.^[^ ^39^ ^]^ This observation is highly relevant and could be a plausible reason for the selective degradation of Sn‐rich areas, assuming that Bi gets much more mobile in Mg_2_Sn than in Mg_2_Si (Bi has a radius comparable to Sn instead of Si). This offers a further potential explanation for the observed selective Mg(OH)_2_ layer formation: the Sn‐rich matrix contains a relatively high Bi‐related defect density while the Si‐rich islands have no or less Bi‐related defects.^[^ ^28^ ^]^

In polycrystalline samples as studied here, lattice diffusion and grain boundary diffusion play a role, with the latter usually being the faster transport mechanism.^[^ ^76^ ^]^ However, studies on this aspect for Mg_2_(Si,Sn) are scarce and partially conflicting. Wang et al.^[^ ^76^ ^]^ have investigated the “growth” of Mg_2_Si showing that it is largely governed by bulk diffusion, with the effect of grain boundary diffusion being negligible. Kogut et al.^[^ ^77^ ^]^ have shown that in the initial stages of Mg_2_Si growth on thin films, the transport mechanism is thermally driven by bulk interstitial Mg diffusion into the Si substrate and then, with increase of temperature, the grain boundaries are leading to acceleration of the diffusion. Thus, this indicates a mixed diffusion phenomenon during the synthesis of these specimens. Lastly, if one looks at Figure 3d,e, one cannot see any Mg(OH)_2_ decoration at the grain boundaries (≈5 μm) but the whole surface is covered by the nonprotective layer. Seeing Mg(OH)_2_ decoration only at the grain boundaries would have been a sign for the more important role of grain boundary diffusion over bulk diffusion. However, it could also be that Mg reaches the surface through grain boundaries mainly and then distributes over the sample surface before reacting with moisture from the atmosphere. Hence, while it is clear from experimental findings and first‐principles calculations that bulk diffusion of Mg is fast in Mg_2_(Si,Sn), evidence of the impact of grain boundary diffusion requires further studies.

Similar material degradation was also observed by Li et al.^[^ ^69^ ^]^ and Wu et al.^[^ ^78^ ^]^ on Mg_3_Sb_2_ material, showing Mg diffusion to be a big challenge for Mg‐based materials stored in ambient conditions. For Mg_2_(Si,Sn) material, one way to avoid the degradation process would be to store the material in a dry and inert atmosphere. In those conditions, humidity and Mg(OH)_2_ formation would be avoided. Another solution could be the use of coatings as suggested by Zhang et al.^[^ ^79^ ^]^ Yin et al.^[^ ^38^ ^]^ and Skomedal et al.^[^ ^80^ ^]^. Finally, defect engineering to reduce the Mg vacancy density would offer a more fundamental path to slow the diffusion down to acceptable levels.

## 4 Conclusions

In this article, we present the degradation mechanism of Mg_2_(Si,Sn) n‐type solid solution by investigating Mg diffusion at RT. The p‐ and n‐type Mg_2_(Si,Sn) samples’ TE properties were reassessed by integral measurement after being stored at RT in ambient atmosphere for 2 years. It appears that p‐type material remains stable while n‐type drastically deteriorates leading to a decreased charge carrier concentration from 3.3 × 10^20^ to 2.2 × 10^20^ cm^−3^, microstructural changes, substantial Mg diffusion, and surface oxidation (formation of Mg(OH)_2_ at the surface on Sn‐rich phases exclusively). The main difference between n‐ and p‐type materials is that there is no excess Mg in p type; therefore, no Mg is lost from the material by diffusion. In contrast, we show that the observed n‐type degradation is linked to Mg loss via diffusion inside and out of the material, which shows a high selectivity to the Si to Sn ratio. According to experimental results and first‐principles calculations, we deduced that the Mg diffusion in the lattice is fast (approximately millimeter per year at RT), mediated by highly mobile Mg vacancies which have a larger density in Mg_2_Sn than in Mg_2_Si. All in all, we deduce that the material degradation is driven by the following mechanism: Mg lattice diffusion from the matrix to the surface reacting with moisture forming a non‐protective layer of Mg(OH)_2_ over the solid solution. Even though many actually employed strategies (nanostructuring, alloying, grain boundary engineering) lead to performance improvement of the TE materials, they might also affect their stability, requiring thorough research in this direction.

## 5 Experimental Section

5.1

5.1.1

##### Materials Synthesis

n‐type Mg_2.06_Si_0.3_Sn_0.665_Bi_0.035_ and p‐type Mg_1.97_Li_0.03_Si_0.3_Sn_0.7_ powder materials were synthesized following the melting route reported in several publications^[^ ^43^, ^44^, ^81^ ^]^ from our group. N‐type was synthesized with 2 at% Mg‐excess to compensate for the Mg‐loss, which occurs while melting and then sintering the powders. Pellets were sintered by a direct current sinter press (DSP 510 SE, Dr. Fritsch GmbH) in vacuum (≈10^−5^ bar) at a temperature of 973 K for 20 min for n‐type and 10 min for p‐type, under an external pressure of 66 MPa on the die with a heating rate of 1 K s^−1^ to obtain compacted pellets. Samples’ density was determined using Archimedes method with an error uncertainty of around 5%. The mass density of n‐ and p‐type samples both before and after aging did not change within measurement uncertainty and can be found in Table S1, Supporting Information. The samples were then characterized and stored in a drawer in ambient conditions at standard temperature and pressure of 298 K and 1 bar, respectively. The samples were stored in an uncontrolled environment, which means that the atmosphere could have an influence on the degradation mechanism. During the first year of aging, the n‐type sample was stored in a drawer in air at RT, protected by a graphite spray layer, which was essential for the thermal conductivity measurement. Note that this graphite layer was not removed following the thermal conductivity measurement. It was then removed once the transport properties were reevaluated after 1 year of storage in air at RT. The sample was finally stored, again in air at RT, uncoated, for another year in the drawer before being remeasured one last time, after a total storage time of 2 years.

Diffusion couples were prepared by joining Mg_1.95_Si_0.3_Sn_0.7_ and Mg_1.95_Si pellets with Mg foils at 823 K. The joining process was executed in a sinter press in vacuum under the influence of a direct current for 20 min. Nominally Mg‐deficient material was used for the diffusion couples experiments to guarantee that the Mg content in the sample was close to the lower solubility limit, i.e., in the thermodynamically Mg‐poor state. This caused the largest difference in Mg chemical potential between the two parts of the diffusion couple, facilitating Mg diffusion from the foil into the TE material. The magnesium poor samples were synthesized employing a high‐energy mechanical alloying mill (SPEX 8000D Shaker Mill) with stainless steel jars and balls for 4 h, followed by a sintering step. The sintering step was the same as detailed in the previous paragraph.

##### Characterization

The samples’ microstructure and phase purity were characterized by backscattered electron images using a ZEISS ultra 55 SEM device, equipped with an Oxford EDX detector (ultim max 100). AFM was used to check the sample surface height differences and material surface topography before and after degradation. XRD pattern of the n‐type sample was obtained using a Bruker D8 device with secondary monochromator, Co–K_*α*_ radiation (1.78897 Å), and step size 0.01° in the *2θ* range (20°–80°), as shown in Figure S4, Supporting Information.

##### TE Measurements

The samples’ functional homogeneity at RT was checked by a spatial mapping of the Seebeck coefficient using an in‐house developed transient potential and Seebeck microprobe (TPSM)^[^ ^54^, ^82^ ^]^ and a PSM.^[^ ^54^, ^56^, ^82^ ^]^ Both devices followed the same measurement principle but differed in their spatial resolutions (3–5 μm for the TPSM and ≈50 μm for the PSM). It consisted of a fine heated microprobe travelling across the dimension of the material, which locally heated the surface of the sample. The local heat resulted in a temperature gradient across the probe inducing a voltage from which the Seebeck coefficient can be determined.^[^ ^83^ ^]^ The Seebeck coefficient values obtained using a PSM were known to be underestimated compared to those obtained by integral measurement using our in‐house *Sσ* measurement device. The variation in temperature between the thermocouple junction's effective position and the point where the thermovoltage was gauged was responsible for this phenomenon. Consequently, there was an experimentally determined disparity of approximately 10–20% from the recorded Seebeck values in the PSM. This phenomenon was also recognized as the cold finger effect.^[^ ^55^, ^84^ ^]^

The temperature‐dependent electrical conductivity (*σ*) and Seebeck coefficient (*S*) were measured using an in‐house developed device with a four‐probe technique under helium atmosphere.^[^ ^51^, ^52^ ^]^ The thermal diffusivity (*α*) measurement was performed using a laser flash method (Netzsch LFA 427 apparatus). From this, the thermal conductivity (*κ*) shown in Figure S1, Supporting Information, was calculated using the following relation: *κ* = *αρC*_p_, where *ρ* and *C*_p_ are the sample density and heat capacity dependent on the composition at constant pressure, respectively. *C*_p_ was calculated using the Dulong–Petit limit estimating the specific heat at constant volume $(c_{V}^{\text{DP}}):\;C_{P}=c_{V}^{\text{DP}}+\frac{9E_{t}^{2}T}{\beta_{T}\rho }$, $E_{t}^{{\text{Mg}}_{2}{\text{Si}}_{0\text{.3}}{\text{Sn}}_{0\text{.7}}}\approx 1.8\times 10^{-5}K^{-1}$, and $\beta_{T}^{{\text{Mg}}_{2}{\text{Si}}_{0\text{.3}}{\text{Sn}}_{0\text{.7}}}\approx 2.19\times 10^{-11}{\text{Pa}}^{-1}$, where *E*_t_ and *β*_t_ are the linear coefficient of thermal expansion and isothermal compressibility of Mg_2_Si_0.3_Sn_0.7_, respectively. Measurements were performed in He and Ar from 300 to 723 K. Measurement error uncertainties for *S*, *σ*, and *κ* were ±5%, ±5%, and ±8%, respectively.^[^ ^51^, ^52^ ^]^ The carrier concentration *n* was estimated using the RT‐measured Seebeck coefficient and the Pisarenko plot (see Figure S6, Supporting Information) calculated with an SPB model for a constant effective mass $m_{D}^{*}=2.7m_{0}$.^[^ ^53^ ^]^ Indeed, for this material system, SPB was shown to work relatively well,^[^ ^35^, ^50^, ^85^ ^]^ particularly at RT as employed here. The charge carrier mobility *μ* was then predicted using the estimated charge carrier concentration and the measurement electrical conductivity following the relation $\mu =\frac{\sigma }{ne}$.

##### First‐Principles Calculations

First‐principles calculations were performed to investigate the diffusion properties of defects in Mg_2_Si and Mg_2_Sn, within hybrid‐density‐functional theory.^[^ ^86^, ^87^, ^88^ ^]^ We used the generalized‐gradient approximation exchange–correlation functional with the Perdew–Burke–Ernzerhof parameterization,^[^ ^89^ ^]^ the projector‐augmented‐wave pseudopotentials,^[^ ^90^, ^91^ ^]^ and the Heyd‐Scuseria‐Ernzerhof (HSE) screened hybrid exchange correlation functional (HSE06; mixing parameter of 25% for the exact Hartree–Fock exchange and the screening parameter of 0.208 Å^−1^),^[^ ^1^, ^88^ ^]^ as implemented in the Vienna ab initio simulation package code.^[^ ^91^ ^]^ For structural model, we used the experimental lattice parameters of 6.35 and 6.75 Å for Mg_2_Si and Mg_2_Sn, respectively. The used calculation setting was the same as far the previous work of Ayachi et al.^[^ ^67^ ^]^

For diffusion of defects, we used the cNEB method to calculate the energy barrier of defect migration.^[^ ^92^ ^]^ The diffusion constant *D* was calculated as $D=a^{2}C_{v}\Gamma$,^[^ ^93^ ^]^ where a is the lattice jump distance of the defect and *C*_ν_ is the defect concentration of a related‐vacancy defect for self‐diffusion. However, in the case of vacancy and interstitial defects, *C*_ν_ was equal to 1. The transition rate Γ was calculated from the vibration frequency of defect at the ground and saddle point, and the defect migration energy *E*_mig_. Here, the effective frequencies of defects were calculated from the Γ‐point phonon vibration modes of a defective supercell within the density‐functional perturbation theory.^[^ ^94^ ^]^ For charged defect formation energies of intrinsic defects, please refer to the previous reports.^[^ ^46^, ^67^ ^]^

## Conflict of Interest

The authors declare no conflict of interest.

## Supporting information

Supplementary Material

## Data Availability

The data that support the findings of this study are available in the supplementary material of this article.
