# Supplementary material for: Instability Mechanism in Thermoelectric Mg2(Si,Sn) and the Role of Mg Diffusion at Room Temperature
Source: Small Sci. 2024 Feb 14;5(3):2300298. doi: 10.1002/smsc.202300298 (PMC12245083; doi:10.1002/smsc.202300298)
Supplement: Supplementary file 1 — Supplementary Material [file SMSC-5-2300298-s001.pdf]

Supplementary Information

Instability mechanism in thermoelectric $Mg_2(Si,Sn)$ and the role of Mg diffusion at room temperature

Amandine Duparchy[a*], *Radhika Deshpande*[a], *Aryan Sankhla*[a], *Sanyukta Ghosh*[a], *Julia Camut*[a], *Sungjin Park*[b], *SuDong Park*[b], *Byungki Ryu*[b**], *Eckhard Mueller*[ac], *Johannes de Boor*[ad***]

[a] Institute of Materials Research, German Aerospace Center (DLR), D–51147 Cologne, Germany

[b] Energy Conversion Research Center, Electrical Materials Research Division, Korea Electrotechnology Research Institute (KERI), Changwon 51543, South Korea

[c] Institute of Inorganic and Analytical Chemistry, Justus Liebig University Giessen, Heinrich-Buff-Ring 17, D–35392 Giessen, Germany

[d] University of Duisburg-Essen, Faculty of Engineering, Institute of Technology for Nanostructures (NST) and CENIDE, 47057 Duisburg, Germany

Corresponding Author(s): amandine.duparchy@dlr.de*, byungkiryu@keri.re.kr**, Johannes.deBoor@dlr.de***

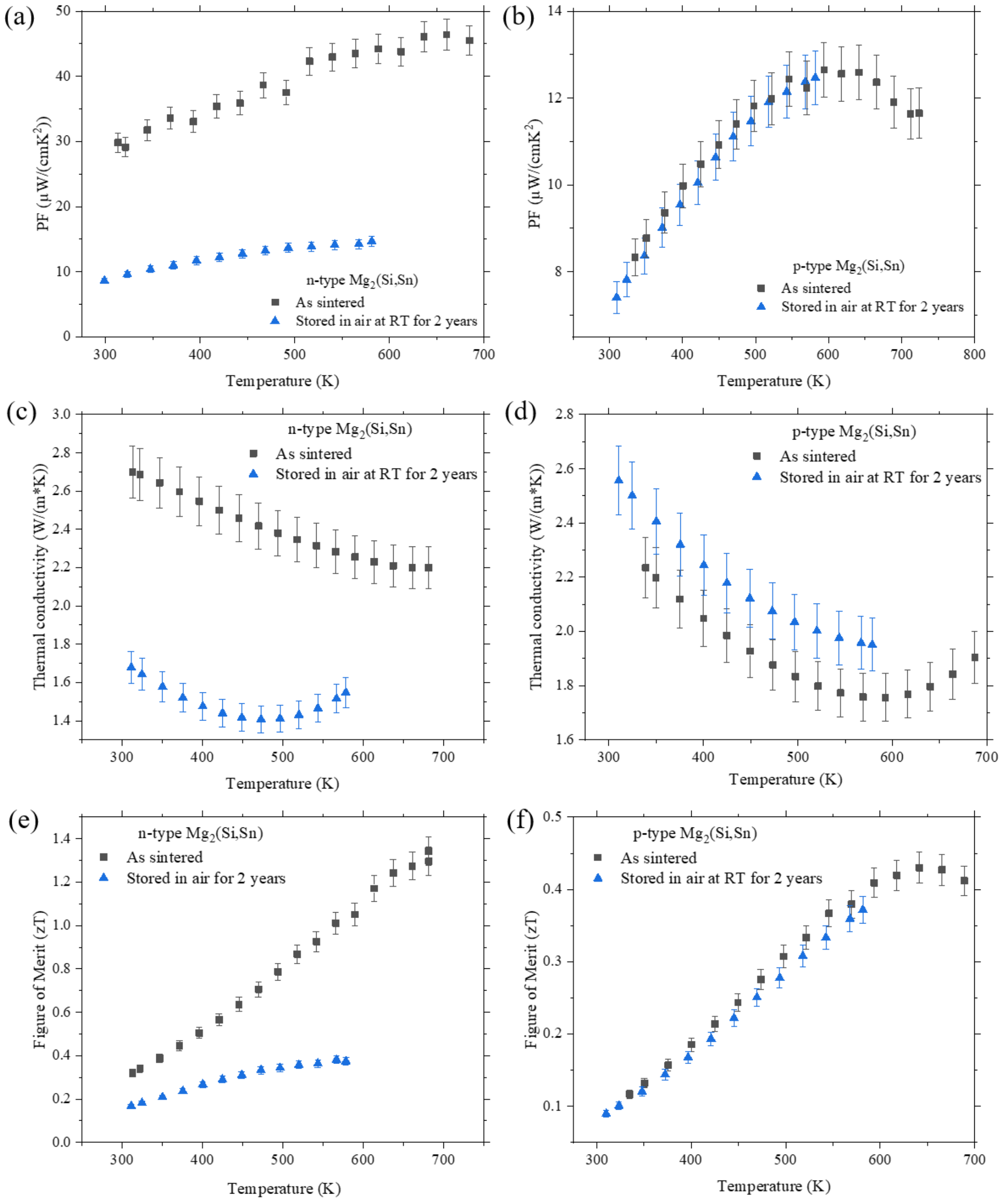

Figure S1. Temperature dependent Power Factor (PF) of the n-type $Mg_2(Si,Sn)$ (a) and p-type $Mg_2(Si,Sn)$; thermal conductivity of n-type (c) and p-type (d) and figure of merit of the n-type (e) and p-type (f) samples after synthesis (gray rectangles) and after being stored in air at room temperature for 2 years (blue triangles) for the n-type and p-type samples. Cooling data are plotted.

Table S1: Densities of n- and p-type $Mg_2(Si,Sn)$ samples as sintered and after aging.

	Density (g/cm^3)	
	As sintered	Aged
n-type	3.134	3.130
p-type	3.137	3.097

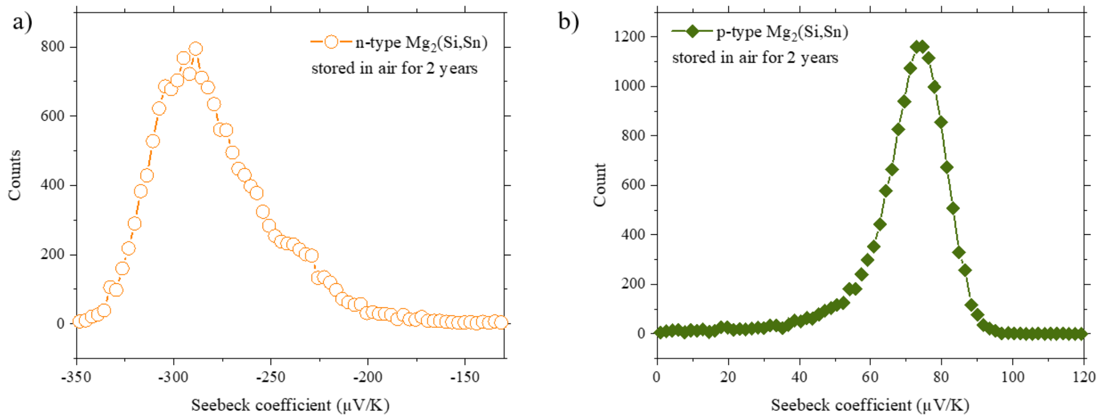

Figure S2. Full width at half maximum (FWHM) of (a) the n-type spatial Seebeck mapping showed in Fig.2a in the main manuscript and (b) the p-type spatial Seebeck mapping showed in Fig.2b in the main manuscript.

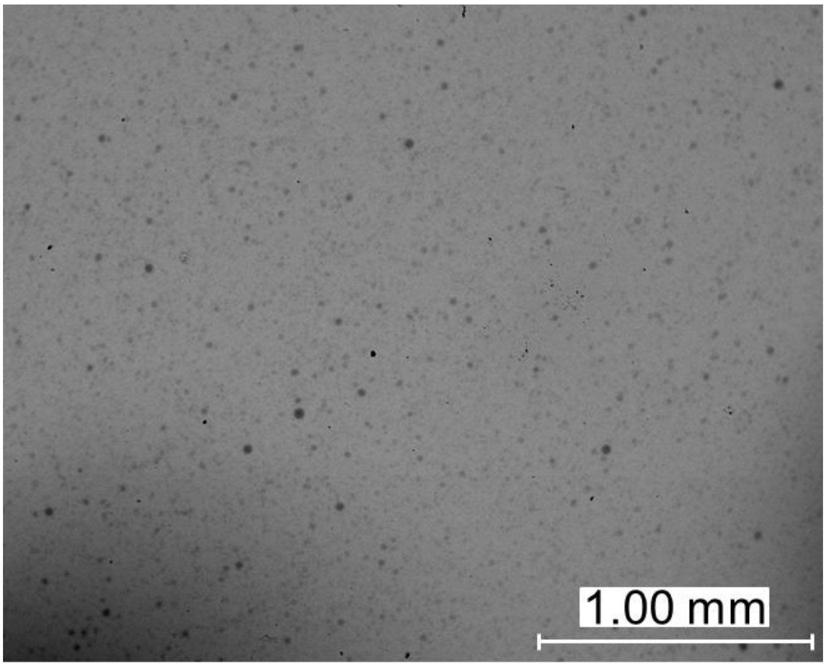

Figure S3. Low magnification SEM image of n-type $Mg_2(Si,Sn)$ sample emphasizing that the material is 98% single phase. The Si-rich islands (black phases) are remnants from the synthesis process.

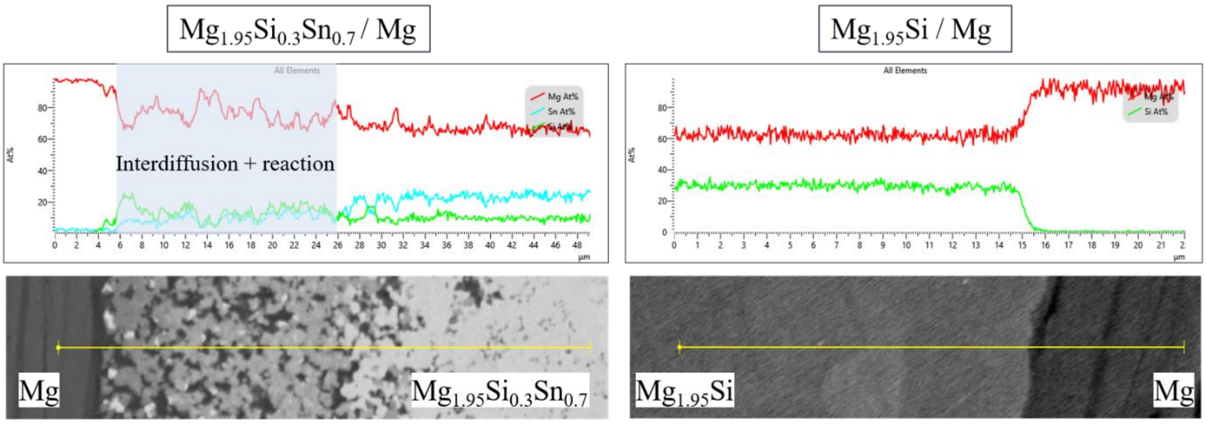

Figure S4. EDX line scan of Mg/Mg$_{1.95}$Si$_{0.3}$Sn$_{0.7}$ and Mg$_{1.95}$Si/Mg diffusion couple interfaces. We can see on the left line scan that an interdiffusion zone formed with a thickness of ~ 20 µm while, on the right side, for the Sn-free Mg$_{1.95}$Si/Mg diffusion couple, the interface is clear with no interdiffusion zone.

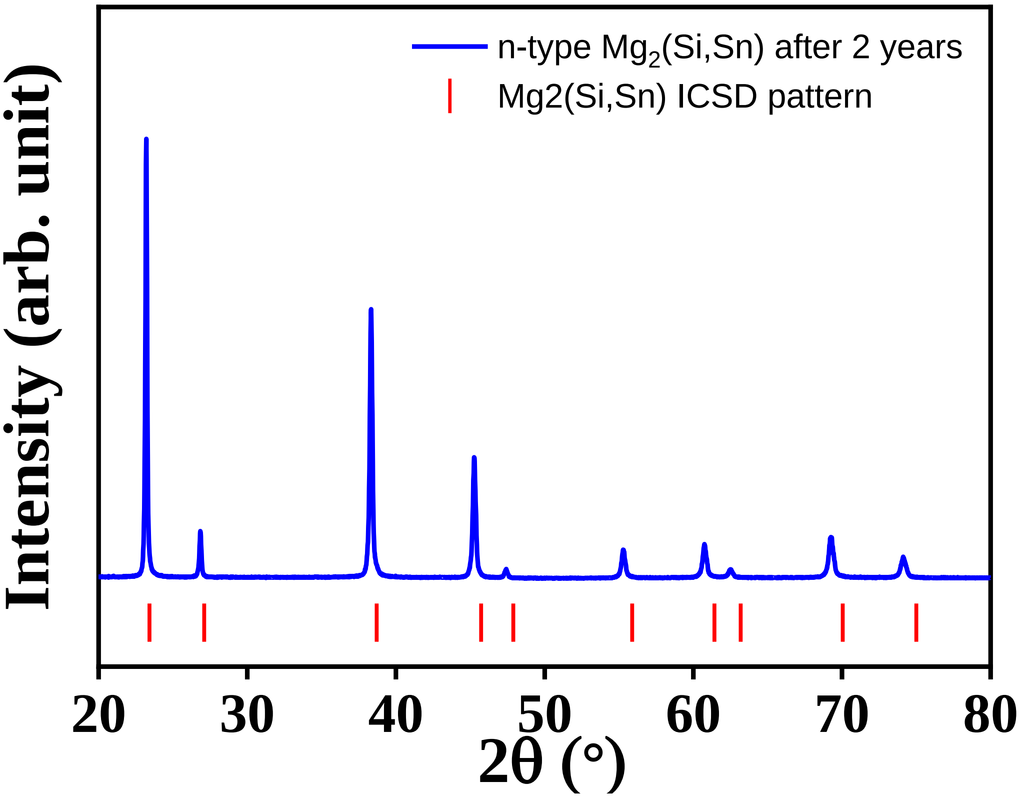

Figure S5. XRD pattern of n-type Mg$_{2.06}$Si$_{0.3}$Sn$_{0.665}$Bi$_{0.035}$ after being stored for 2 years in air at RT. No MgO peak detected. Single phase material.

As shown in Figure S6, the chemical potential was determined using a single parabolic band model which is not valid for Mg_2(Si,Sn) sample above 350 K. Hence, SPB is valid only around room temperature (where it is employed here), represented with dark colors.

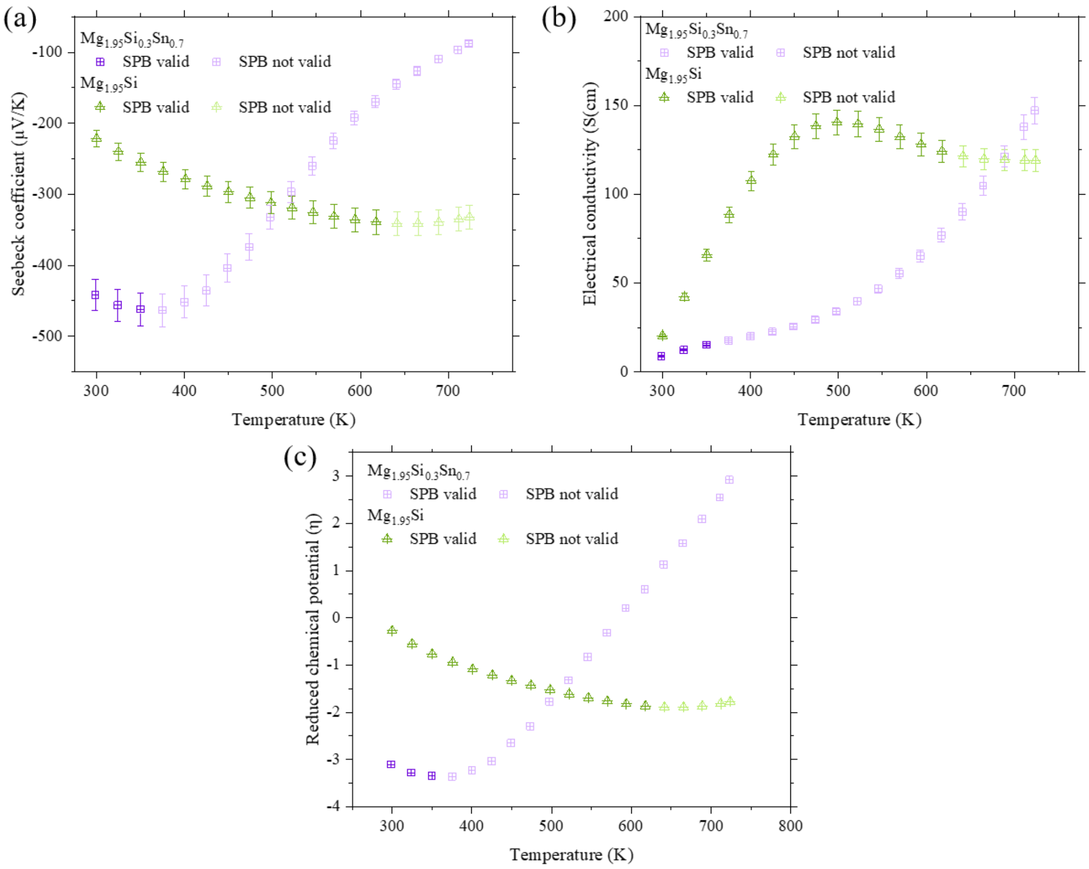

Figure S6. (a) Seebeck coefficient, (b) electrical conductivity and (c) reduced chemical potential of the bulk $Mg_{1.95}Si$ and $Mg_{1.95}Si_{0.3}Sn_{0.7}$ used for diffusion couple experiments. Dark purple and dark green colors represent the data where SPB is valid while light green and light purple represent the data where SPB is not valid.

Table S2: Reduced chemical potential and charge carrier concentration of $Mg_{1.95}Si$ and $Mg_{1.95}Si_{0.3}Sn_{0.7}$ at room temperature determined by SPB.

	Reduced chemical potential	Charge carrier concentration (cm^{-3})
$Mg_{1.95}Si$	-0.3	$4.4*10^{19}$
$Mg_{1.95}Si_{0.3}Sn_{0.7}$	-3.1	$5*10^{18}$

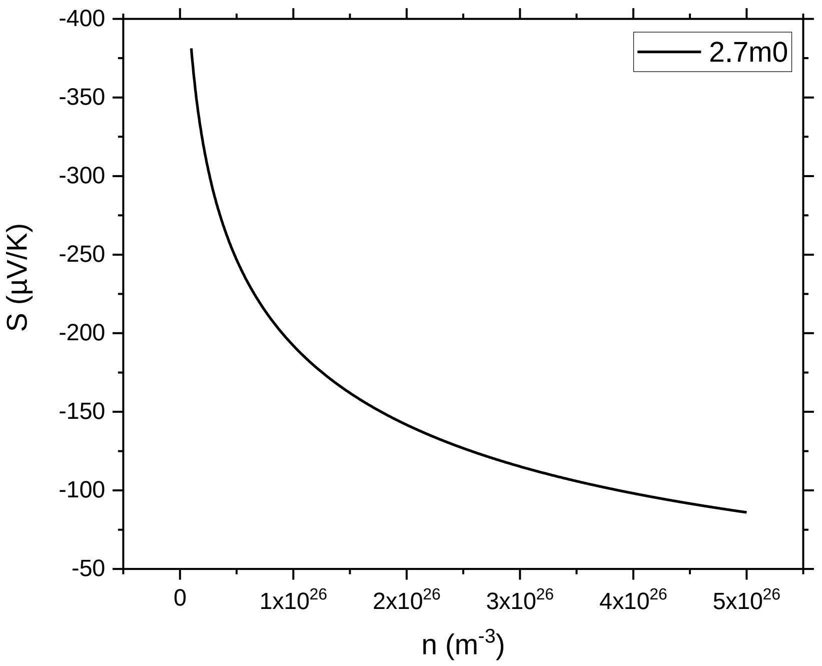

Figure S7. Pisarenko plot of $Mg_2(Si,Sn)$ for $m^*=2.7m_0$
